# Compliance of iron and folic acid supplementation and status of anaemia during pregnancy in the Eastern Terai of Nepal: findings from hospital based cross sectional study

**DOI:** 10.1186/s13104-019-4167-6

**Published:** 2019-03-12

**Authors:** Krishna Deo Yadav, Uday Narayan Yadav, Rajendra Raj Wagle, Dip Narayan Thakur, Sarmila Dhakal

**Affiliations:** 1 Department of Community Medicine and Public Health, Institute of Medicine, Tribhuvan University, Kathmandu, Nepal; 2 Ministry of Social Development, Biratnagar, Province One Nepal; 3 Forum for Health Research and Development, Dharan, Nepal; 4 Centre for Primary Health Care and Equity, School of Public Health and Community Medicine, UNSW, Sydney, Australia; 5 Karnali Academy of Health Sciences, Jumla, Nepal; 6 Nepal Public Health Foundation, Kathmandu, Nepal; 7 Nepal Institute of Health Sciences, Kathmandu, Nepal

**Keywords:** Iron and folic acid supplementation, Compliance, Anaemia during pregnancy

## Abstract

**Objectives:**

Our study aimed to assess local data for compliance with IFA supplementation and prevalence of anaemia among the pregnant mothers visiting government health facilities of eastern Nepal.

**Results:**

In our study samples, IFA compliance rate was 58% during pregnancy and 42% were anaemic. Anemia was 24 times more likely to occur in IFA noncompliant women during pregnancy than their counterparts (aOR = 24.2, 95% CI 10.1–58.3), and anemia was three times less likely to be found in those taking foods rich in heme–iron than their counterparts (aOR = 3.3, 95% CI 1.4–8.1).

## Introduction

Iron deficiency is one of the most prevalent nutrient deficiencies in the world [1]. Evidence shows the requirement of iron increases significantly during second and especially during third trimester of pregnancy [2]. During such conditions, dietary iron intake in the majority of population of the developing countries is not sufficient [3]. This could be due to the low consumption of limited animal source food, green leafy vegetables and fruits in their daily life [4], and high utilization of iron for oxygen supply to both mother and fetus [5].

Iron deficiency anaemia is responsible for at least half of the anaemia burden during the pregnancy worldwide [6]. Besides anaemia, Iron deficiency is associated with low birth weight and increased risk of developing perinatal infection and pre-eclampsia, resulting into maternal and perinatal mortality. It may also affect growth and development in utero and in the long term [6, 7]. Similarly, folate deficiency during pregnancy also causes anaemia in women and neural tube defects in foetus [8]. To tackle this, World Health Organisation (WHO) has recommended daily IFA supplementation from second trimester aimed at improving their haemoglobin concentrations and iron status and reduce the risk of anaemia [6].

Despite IFA supplementation being a prioritized program in Nepal since 1980s, anaemia during pregnancy is still an existing public health problem and is noted to be above 40% [9, 10]. The terai/plain region of Nepal which holds more than 50% of the population has made some progress in resolving this problem, despite having IFA supplementation Program. The evidence from Nepal have showed that prevalence of anaemia is higher in Terai as compared to another ecological region of Nepal [10–12]. This potentially suggests that there could be some underlying factors either with IFA supplementation or may be with dietary pattern of the people of this ecological origin. There is dearth of evidence that looked into compliance of IFA and status of anaemia among the pregnant mothers visiting government health facilities. In light of this, our study aimed to assess local data for compliance with IFA supplementation and prevalence of anaemia among the pregnant mothers visiting government health facilities of eastern Nepal.

## Main text

### Methods

This study was hospital based analytical cross-sectional type, conducted between August and February 2016. This study was conducted at three zonal hospitals (Mechi, Koshi and Sagarmatha Zonal Hospitals) and two district hospitals (Sunsari and Siraha district hospitals), where patients of those setting visits for seeking health services. Postpartum mothers having baby of less than 7 days were the study population for this study. Sample size was calculated by using a formula, n = z^2^pq/d^2^ where z = 1.96, p = 38.4% (IFA compliance), q = 61.6%. d = 5.5% (allowable error) and non-response rate of 15% were taken into consideration [12–14].

Of calculated 345 samples, we achieved sample size of 328 with nearly 5% non-response rate. The study participants were enrolled from each hospital using fixed proportionate method using the total deliveries of fiscal year 2015/16. Simple random sampling technique was used to recruit the study samples from postpartum registers of respective hospitals. Data were collected using face to face interview of the eligible participants and clinical variables were recorded from the individual patient file.

Validity and reliability of the study was ensured through development of concrete research proposal and adoption of tools already used in Nepal [10, 15]. Pretesting of tool was performed at Tribhuvan University Teaching Hospital, Kathmandu and Sagarmatha Zonal Hospital, Rajbiraj and needful modification was done prior to field work. The data was collected by one of the members of this team who was fluent in both Nepali and local Maithili language, and was well trained on quality assurance and quality control for conducting field research. Antenatal check-up card and or prescription slip of postpartum mothers was also used for cross verification of the information collected.

IFA intake of at least 80% of the recommended dose (i.e. 144 or more tablet out of recommended 180 or equivalent dose for capsule or liquid IFA) during pregnancy was considered as cut-off for compliance in this study [15]. Similarly, haemoglobin (Hb) level of less than 11.0 gm/dl was considered anaemic [7].

Mothers who didn’t had haemoglobin report of 1 month from the date of delivery, mothers who were diagnosed with severe chronic disease or mental illness and, mothers who had still birth were excluded from the study.

Ethical approval for the study was obtained from Institutional Review Board, Institute of Medicine, Kathmandu, Nepal (Reference number: 97(6-11-E)2-073/7). Written consent was obtained from each participant, and for the underage participants (< 18 years) consent was obtained from a guardian on behalf of any participants prior to interview.

The statistical analyses were performed by International Business Machine-Statistical Package for Social Sciences (IBM-SPSS) version 21. Binary logistic regression model was applied to assess association between dependent and independent variables.

### Results

#### Socio-demographic characteristics and IFA compliance

Mean age of postpartum mothers was 23 (± 3.98) years. Majority of the study samples were ascribed to Hindu religion (86.9%). Similarly, two-third of the mothers were the rural residents at the time of survey. One-fourth of postpartum mothers were illiterate. Home making (87.8%) was the major occupation and almost half of the respondents (47%) were from poor family background.

IFA compliance rate was 58% which differed significantly by ethnicity, religion, literacy of women and their husband, occupation of women and their husband as well as household’s economic status (Table 1).

**Table 1:** Sociodemographic characteristics and IFA compliance

Characteristics	Non-compliance	Compliance	*p* value
Ethnicity			
Mountain and hills caste	13 (28.3)	33 (71.7)	0.045*
Terai/Madhesi caste	124 (44.0)	158 (56.0)	
Age in years			
15–19	26 (49.1)	27 (50.9)	
20–29	96 (39.2)	149 (60.8)	0.264
≥ 30	15 (50.0)	15 (50.0)	
Gravida			
Primi	51 (37.0)	87 (63.0)	0.132
Multi	86 (45.3)	104 (54.7)	
Religion			
Hindu	113 (39.6)	172 (60.4)	
Muslim	21 (60.0)	14 (40.0)	0.024*
Type of residence			
Rural	96 (42.1)	132 (57.9)	
Urban and semi-urban	41 (41.0)	59 (59.0)	0.852
Literacy of women			
Literate	92 (37.2)	155 (62.8)	0.004*
Illiterate	45 (55.6)	36 (44.4)	
Occupation of women			
Housewife (homemaker)	113 (39.2)	175 (60.8)	0.013*
Others	24 (60.0)	16 (40.0)	
Literacy of husband			
Literate	102 (37.2)	172 (62.8)	< 0.001*
Illiterate	35 (64.8)	19 (35.2)	
Occupation of husband			
Daily wages	52 (61.9)	32 (38.1)	< 0.001*
Others	85 (34.8)	159 (65.2)	
Economic status			
Non-poor	43 (27.4)	114 (72.6)	< 0.001*
Poor	94 (55.0)	77 (45.0)	

Figures in parenthesis denotes row percentage, *denotes statistically significant at 5% level of significance

#### Description of anaemia and related factors

Overall, 42% of the postpartum mothers were found to be anaemic during their last pregnancy. Of them, 29.3% suffered from mild anaemia, 12.2% had moderate anaemia and the rest with severe anaemia. Disaggregated findings showed that 23% from mountain and hill origin castes, 49% from Terai/Madhesi dalit origin, 42% from Terai/Madhesi Janjati, 47% of Muslim and 45% of other Terai/Madhesi caste were anaemic.

Besides IFA supplementation, data were also gathered regarding most common etiological factors of anaemia such as history of infection, deworming for worm infestation, intake of iron rich food, intake of iron absorption inhibitors, vitamin A rich food intake, smoking and alcohol consumption as well as history of chronic diseases.

Of total, 91% respondents reported that they had taken the de-worming tablet (Albendazole) and 15% reported fever during their last pregnancy. Similarly, nine respondents (2.7%) had faced some sort of vision problem in the evening/night and three faced vaginal bleeding although they didn’t require any blood transfusion. No respondents reported the presence of chronic diseases ever. Regarding food habit, 86% were non-vegetarian and, the rest were lacto vegetarian. Almost 80% respondents were unable to state at least one iron rich food source. None of the respondents reported tobacco or alcohol consumption in their last pregnancy.

#### Factors affecting status of anaemia

Bivariate regression analysis of factors affecting anaemia is presented in Table 2.

**Table 2:** Bivariate regression analysis for factors affecting status of anemia during pregnancy

Characteristics	No-anemia	Anemia	p-value	COR	95% CI	
					Lower	Upper
Age in years						
15–19	28 (54.9)	23 (45.1)	0.488	1		
20–29	143 (60.3)	94 (39.7)		0.800	0.435	1.473
≥ 30	14 (50.0)	14 (50.0)		1.217	0.483	3.066
Ethnicity						
Mountain and Hill castes	34 (77.3)	10 (22.7)	0.007	1		
Terai/Madhesi castes	151 (55.5)	121 (45.5)		2.725*	1.294	5.736
Birth gap in years						
< 3	51 (49.0)	53 (51.0)	0.019	2.078*	1.124	3.845
≥ 3	50 (66.7)	25 (33.3)		1		
Food habit						
Non-vegetarian	161 (59.0)	112 (41.0)	0.696	0.879	0.459	1.681
Lacto-vegetarian	24 (55.8)	19 (44.2)		1		
IFA compliance						
No	24 (18.5)	106 (53.9)	< 0.001	28.443*	15.433	52.422
Yes	161 (86.6)	25 (13.4)		1		
Albendazole taken						
Yes	170 (59.4)	116 (40.6)	0.318	1		
No	15 (50.0)	15 (50.0)		1.466	0.690	3.114
Fever history						
Yes	19 (42.2)	26 (57.8)	0.016	2.163*	1.141	4.103
No	166 (61.3)	105 (38.7)		1		
Heme iron intake						
Yes	118 (63.1)	69 (36.9)	0.048	1		
No	67 (51.9)	62 (48.1)		1.583*	1.003	2.496
Non-heme iron intake						
Yes	147 (64.8)	80 (35.2)	< 0.001	1		
No	38 (42.7)	51 (57.3)		2.466*	1.495	4.068
Intake of sour foods						
Yes	50 (68.5)	23 (31.5)	0.049	1.739	0.999	3.029
No	135 (55.6)	108 (44.4)		1		
Intake of yellow fruits						
Yes	33 (73.3)	12 (26.7)	0.032	2.153*	1.066	4.348
No	152 (56.1)	119 (43.9)		1		
Tea/coffee						
Taking	103 (58.5)	73 (41.5)	0.993	1.002	0.639	1.572
Not taking	82 (58.6)	58 (41.4)		1		

Figures in parenthesis denotes row percentage, ‘1’ denotes Reference category, *denotes statistically significant

Multivariate analysis using binary logistic regression was carried out to test the effect of IFA compliance on anaemia by adjusting other variables which were significant in univariate analysis (Table 3).

**Table 3:** Multivariate regression analysis for factors affecting status of anemia during pregnancy

Characteristics	(95% CI)			95% CI		
	COR	Lower	Upper	AOR	Lower	Upper
IFA compliance						
No	28.443	15.433	52.422	24.162*	10.009	58.327
Yes	1					
History of fever						
Yes	2.163	1.141	4.103	2.980	0.890	9.981
No	1					
Heme iron intake						
Yes	1					
No	1.583	1.003	2.496	3.347*	1.378	8.133
Non-heme iron intake						
Yes	1					
No	2.466	1.495	4.068	1.462	0.582	3.671
Intake of yellow fruits						
Yes	1					
No	2.153	1.066	4.348	1.163	0.313	4.319
Birth gap in years						
< 3	2.078	1.124	3.845	1.603	0.690	3.728
≥ 3	1					
Ethnicity						
Mountain and Hill castes	1					
Terai/Madhesi castes	2.725	1.294	5.736	2.058	0.589	7.183

Figures in parenthesis denotes row percentage, ‘1’ denotes Reference category, *denotes statistically significant

### Discussion

This study showed that 42% of the women were anaemic during last pregnancy in the Eastern Terai which was 6% less than NDHS (National Demographic Health Survey) 2011, same as that of NDHS 2006 whereas 14% higher than a study carried out by Nepal Health Research Council in 2015 in Mid-western Terai of Nepal [9, 10, 16]. These differences could be because of hospital based setting in our study and geographical setting in previous studies.

Similarly, overall IFA compliance rate was 58%, which was 20% more than findings of NDHS, 2011 and 36% more than findings reported by Mitra Samaj in 2015 [10, 17]. Out study setting could have been attributed to this increment. However, compliance shown by our study was seven percent less as par with findings from the study conducted in South India by Mitra et al. [19], and is similar to findings from Egypt by Ibrahim et al. [18, 19]. These changes could be due to differently operationalization of compliance. This study has considered 80% or above intake of recommended dose as compliance whereas Mitra et al. had considered noncompliance as ‘missing more than 2 doses consecutively’ and Ibrahim et al. implied compliance as ‘intake of 65% or above of recommended dose’. Notably, there was country specific variation in recommended dose and duration of IFA supplementation [19]. WHO has recommended daily intake of 30–60 mg elemental iron during pregnancy starting from second trimester of pregnancy, however India was adopting 100 mg elemental iron for 100 days [6, 19].

Results from NDHS 2013 showed higher compliance among the Mountain and Hill castes than those of Terai/Madhesi castes as well as higher compliance among Hindus in compared to Muslims, which is in line with our findings [12]. Findings also showed that those who were non-compliant to IFA were 24 times more likely to be anaemic. This was supported by retrospective cohort study findings from Saudi Arabia and the randomized community intervention trial study conducted by Dreyfuss et al. in Sarlahi, Nepal in 2000 [20, 21].

Similarly, findings also showed that those who were not taking heme iron rich food during pregnancy were three times more likely to be anaemic than their counterparts. This was supported by a study undertaken by Mahajani et al. [22] in Rajasthan, India which showed that mean haemoglobin level of non-vegetarian was higher (12.07 ± 1.08 g/dl) than those of vegetarian group (10.09 ± 0.95 g/dl).

Our study found that anaemia was 24 times more likely to be found in those women who were noncompliant to IFA supplementation during pregnancy than their counterparts. Similarly, intake of heme iron rich food was seen to be protective. Anaemia was three times less likely to be found in those women who were taking heme iron rich food during pregnancy than those who were not consuming it. Therefore, to combat anaemia during pregnancy in the Eastern Terai of Nepal, IFA supplementation programme needs to be strengthened combined with dietary improvements, micronutrient supplementation, and food fortification to address nutritional anaemia which might in visible change in the target population.

### Limitations

Like other studies, this study does have some limitations. First, changes of recall bias could not be ignoring as mother had to recall practice of last 6 months at the time of interview. Second, the study samples were enrolled from hospital-based setting, compliance could be different than that of community-based study. Similarly, findings could not be generalised to community or primary health centre settings.

## Abbreviations

IFA: iron and folic acid
WHO: World Health Organization
NDHS: Nepal Demography and Health Survey
COR: crude odds ratio
AOR: adjusted odds ratio
CI: confidence interval
